# Proliferation-Related Activity in Endothelial Cells Is Enhanced by Micropower Plasma

**DOI:** 10.1155/2016/4651265

**Published:** 2016-12-12

**Authors:** Kotaro Suzuki, Daisuke Yoshino

**Affiliations:** ^1^Graduate School of Engineering, Tohoku University, 6-6-01 Aramaki-Aoba, Aoba, Sendai 980-8579, Japan; ^2^Institute of Fluid Science, Tohoku University, 2-1-1 Katahira, Aoba, Sendai 980-8577, Japan

## Abstract

Nonthermal plasma has received a lot of attention as a medical treatment technique in recent years. It can easily create various reactive chemical species (ROS) and is harmless to living body. Although plasma at gas-liquid interface has a potential for a biomedical application, the interactions between the gas-liquid plasma and living cells remain unclear. Here, we show characteristics of a micropower plasma with 0.018 W of the power input, generated at gas-liquid interface. We also provide the evidence of plasma-induced enhancement in proliferation activity of endothelial cells. The plasma produced H_2_O_2_, HNO_2_, and HNO_3_ in phosphate buffered saline containing Mg^++^ and Ca^++^ (PBS(+)), and their concentration increased linearly during 600-second discharge. The value of pH in PBS(+) against the plasma discharge time was stable at about 7.0. Temperature in PBS(+) rose monotonically, and its rise was up to 0.8°C at the bottom of a cell-cultured dish by the plasma discharge for 600 s. Short-time treatment of the plasma enhanced proliferation activity of endothelial cells. In contrast, the treatment of H_2_O_2_ does not enhance the cell proliferation. Thus, the ROS production and the nuclear factor-kappa B (NF-*κ*B) activation due to the plasma treatment might be related to enhancement of the cell proliferation. Our results may potentially provide the basis for developing the biomedical applications using the gas-liquid plasma.

## 1. Introduction

Plasma is called the fourth state of matter following solid, liquid, and gas, and it is composed of charged particles, excited particles, chemically reactive species, and neutral particles. Recently, plasma has been developed for a wide range of medical applications such as sterilization [1, 2], surface modification of a medical equipment [3], and blood coagulation [4]. They are generically known as “plasma medicine” [5]. The plasma used for medical applications is classified with two types [6]: one is thermal plasma whose temperature is around 10^4^ K, and the other is nonthermal plasma whose temperature is around room temperature. Nonthermal plasma sources have become popular for medical applications because there is no measurable damage to living tissue.

In recent years, plasma treatments to enhance cell proliferation have attracted attention in the field of plasma medicine, and there have been many researchers about the plasma treatments of living cells [7, 8]. It is believed that reactive oxygen species (ROS) produced by the plasma have a positive effect on the therapeutic actions. However, the interactions between nonthermal plasma and living cells are still unclear. Several types of plasma sources for biomedical applications such as plasma jets and surface discharges are generally used [9]. Plasma at gas-liquid interface also has a potential for the biomedical application because the plasma produces ROS in liquid effectively by a synergistic effect of chemical reactions in gas phase and gas-liquid interface [10–14]. However, the plasma at gas-liquid interface is not used much for the biomedical applications.

This study aims to understand characteristics of a micropower plasma generated at gas-liquid interface and to reveal responses of living cells to the plasma for development of a novel biomedical applications using the plasma. First, we measure characteristics of the micropower plasma, namely, power input and emission spectra of the plasma, changes in pH and temperature in a liquid by the plasma treatment, and production of chemical species in the liquid. Secondly, we evaluate an effect of the plasma treatment on living cells focusing on the cell viability and discuss a mechanism for cellular response to the plasma treatment.

## 2. Materials and Methods

### 2.1. Micropower Plasma Source

**Figure 1:**
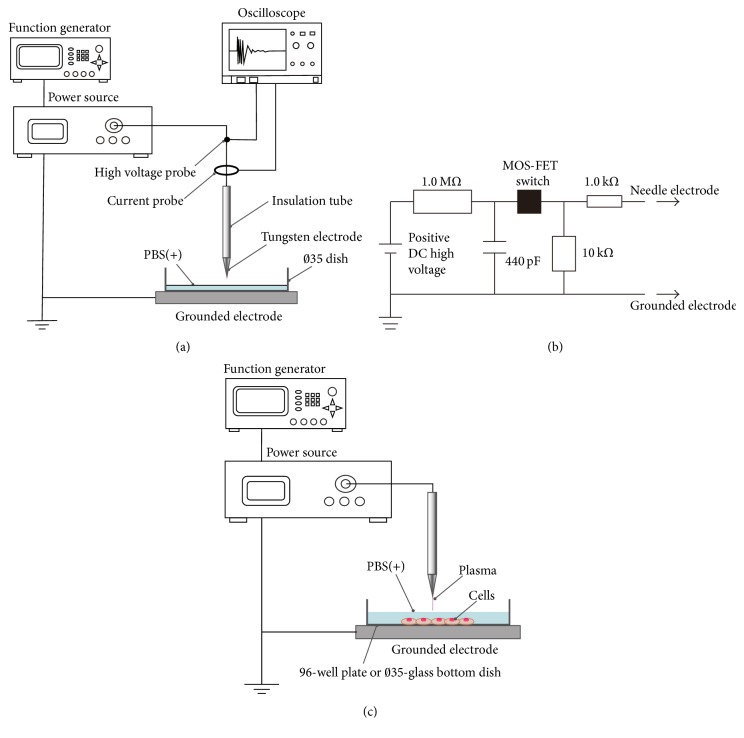
Schematics of the experimental setup (a), electric circuit in the power source (b) to generate micropower plasma, and the experimental setup to expose cells to the micropower plasma (c).

Figure 1 shows the schematics of the experimental setup (a) and the power source (b) to generate micropower plasma. The power source consisted of a direct current (DC) power supply (HGR10-10P, Matsusada Precision Inc.), a metal-oxide-semiconductor field-effect transistor switch (MOS-FET switch; HTS 151-02, BEHLIKE), three resistors (1.0 MΩ, 10 kΩ, and 1.0 kΩ), and a capacitor (440 pF). The MOS-FET switch was controlled by a function generator (FG-274, TEXIO). The applied voltage and discharge current were measured using a high-voltage prove (PHV4-1221, PMK) and a current probe (FCT-028-5.0-WB, Bergoz), respectively. Waveforms were monitored using an oscilloscope (waveRunner 62Xi, Lecroy). A tungsten needle electrode (0.5 mm in diameter) covered with an insulation tube (011, TGK) was set at the center of a 35-mm diameter cell culture dish (3000-035 or 3910-35, Iwaki) or 96-well cell culture plate (353072, BD Falcon). The dish or plate was filled with 2 mL or 40 *μ*L, respectively, of phosphate buffered saline (PBS; 05913, Nissui) containing Mg^++^ and Ca^++^ (PBS(+)). The distance from a liquid surface to the tip of the electrode was 1 mm (for the 35-mm diameter dish) or 0.5 mm (for the 96-well plate), respectively. The rectangular-wave voltage applied to the tungsten needle electrode was +5.5 kV from 0 to peak, with a frequency of 100 Hz and a duty ratio of 50%.

### 2.2. Measurement of Characteristics of Micropower Plasma and Chemical Species

Light emitted from the plasma was captured using a digital camera (D4, Nikon) equipped with a macrolens (AF Micro-Nikkor 200 mm f/4D IF-ED, Nikon). The exposure time was 10 s, ISO sensitivity was 1600, and the diaphragm of the camera was 4. Emission spectra were measured using a multichannel optical spectrometer (PMA-12, Hamamatsu Photonics). The range of measured wavelength was from 200 nm to 860 nm. The distance from the part of plasma discharge to the spectrometer was 5 cm. Temperature of the generated plasma was estimated from the vibrational and rotational temperatures of nitrogen molecule (N_2_), which were calculated with the measured spectral lines of N_2_-second positive system (SPS) and an analysis software [15].

The pH of the solution was measured using a pH meter (twin pH, AS ONE). The temperature of the solution was measured using a thermocouple (T/T-E40-1, Ishikawa Trading) and a temperature controller (E5CN-HQ2, OMRON).

Concentrations of dissolved hydrogen peroxide (H_2_O_2_), nitrous acid (HNO_2_), and nitric acid (HNO_3_) in the solution were measured using a water quality meter (DPM-MT, Kyoritsu Chemical-Check Laboratories). The reagent containing 4-aminoantipyrine (WAK-H2O2, Kyoritsu Chemical-Check Laboratories) or naphthylethylenediamine (WAK-NO2 or WAK-NO3, Kyoritsu Chemical-Check Laboratories) was added into the plasma-treated solution. The mixture of the plasma-treated solution and the reagent was gently shaken ten times, and the color of the mixture was then changed by the reaction between the reagent and the chemicals dissolved in the solution. The colored mixture put into the quality meter.

### 2.3. Cell Culture

Human umbilical vein endothelial cells (HUVECs; 200K-05n, Cell Applications) from the fourth to eighth passages were used for the experiments in this study. HUVECs were cultured in the 35-mm diameter dish or the 96-well plate that were precoated with 0.1% bovine gelatin (G9391, Sigma-Aldrich). HUVECs were cultured in Medium 199 (M199; 31100035, Gibco) containing 20% heat-inactivated fetal bovine serum (FBS; 12483020, Gibco), 10 *μ*g/L human basic fibroblast growth factor (bFGF; GF-030-3, AUSTRAL Biologicals), and 0.1% penicillin/streptomycin (P/S; 15140122, Gibco) (growth medium (GM)). After experiments, HUVECs were maintained in M199 containing 10% heat-inactivated FBS and 0.1% P/S (maintenance medium (MM)).

### 2.4. Cell Viability Assay

HUVECs were seeded into the 96-well plate at 2000 cells/well and then were incubated in the GM for 24 hours. After incubation in the MM for 1 hour, the cells were washed twice with PBS(+) to remove bFGF and FBS. The 96-well plate was filled with 40 *μ*L of PBS(+), and the cells were then treated with the micropower plasma, as illustrated in Figure 1(c). The distance from the liquid surface to the tip of the electrode was 0.5 mm. The plasma irradiation time was 0, 30, 60, 90, 120, 180, 300, or 420 s. The cells were washed twice with the MM immediately after plasma treatment. After 24-hour incubation, the cells were stained with 10 *μ*L/well of the Cell Count Reagent SF (07553-15, Nacalai tesque) for 1 hour. Absorbance of the cells was measured at 450 nm using a microplate reader (Model 680 XR, Bio-Rad). The ratio of live cells was calculated according to the formula: ratio of viable cells = plasma-treated sample/control (nontreated sample).

### 2.5. Measurement of ROS in the Plasma-Treated Cells

HUVECs were cultured in the 35-mm diameter dish to reach 80% confluence. After incubation with 10 *μ*M 2′,7′-dichlorodihydrofluorescein diacetate (DCFH-DA; 35845, Sigma-Aldrich) in the GM for 50 min, the GM was washed out using PBS(+). The dish was filled with 2 mL of PBS(+), and the cells were then treated with the micropower plasma. The distance from the liquid surface to the tip of the electrode was 1 mm. The plasma irradiation time was 0, 60, 180, 420, or 600 s. The cells were washed twice with PBS(+) immediately after plasma treatment. The cells were incubated in the MM for 15 min, and the fluorescent images of ROS in the cells were then observed using an inverted fluorescence microscope (Axio Observer D1; Carl Zeiss). The fluorescent intensity of ROS in the cells was evaluated with the ImageJ software (US National Institutes of Health). On the other hand, the fluorescent intensity of ROS in the plasma-treated cells was also measured using a flow cytometer (guava easyCyte™ HT, Merck Millipore). After plasma treatment, the cells were incubated in the MM for 15 min. The cells were harvested with 0.05% Trypsin-EDTA (25300054, Gibco) and spin down at 1000 rpm for 5 min. The cells were resuspended in 150 *μ*L of PBS(+), and the fluorescent intensity of ROS in 5000 of the cells was measured at 488 nm using the flow cytometer. As a control experiment, the cells were treated with PBS(+) containing H_2_O_2_ (081-04215, Wako Pure Chemical Industries) for 600 s.

### 2.6. Evaluation of Nuclear Factor-Kappa B (NF-*κ*B) Activation

HUVECs were cultured in the 35-mm diameter dish to reach 80% confluence. The cells were washed twice with PBS(+). The dish was filled with 2 mL of PBS(+), and the cells were then treated with the micropower plasma. The distance from the liquid surface to the tip of the electrode was 1 mm. The plasma irradiation time was 300 s. The cells were washed twice with PBS(+) immediately after plasma treatment. The cells were incubated in the MM for 0, 15, 30, and 45 min and then fixed with ice-cold methanol for 10 min at −20°C. The cells were subsequently treated with 0.3% TritonX-100 (17-1315-01, Pharmacia Biotech) and 1% Block Ace (BA; UKB40, DS Pharma Biomedical) for 1 hour at room temperature for membrane permeabilization and blocking of nonspecific adsorption of antibody. They were then incubated in the rabbit NF-*κ*B p65 antibody (1 : 300 diluted in PBS containing 1% BA, sc-372, Santa Cruz Biotechnology) for 1 hour. After washing with PBS, they were incubated in Alexa Fluor 488 goat anti-rabbit secondary antibody (1 : 1000 diluted in PBS, A-11034, Invitrogen) for 2 hours. Cell nucleus was stained with 1 *μ*g/mL 4′,6-diamidino-2-phenylindole (DAPI; D1306, Life Technologies) for 5 min. The fluorescent images of the stained cells were observed using the inverted fluorescence microscope. Localization of NF-*κ*B p65 in the cells was evaluated based on the fluorescent intensity using the ImageJ software. Percentage of NF-*κ*B p65 translocated into the cell nucleus was calculated according to the formula: % of NF-*κ*B p65 translocation (i.e., NF-*κ*B activation) = (fluorescent intensity of NF-*κ*B p65 in the cell nucleus/fluorescent intensity of NF-*κ*B p65 in the whole cell) × 100. As a control experiment, the cells were treated with PBS(+) containing H_2_O_2_ for 300 s. We used catalase (C9322, Sigma-Aldrich) to inhibit influence of H_2_O_2_ generated by the plasma discharge on the cells.

### 2.7. Statistical Analysis

All values are shown as mean ± standard deviation (SD) unless stated otherwise. The statistical significance was determined using an analysis of unequal variances two-tailed *t*-test (Welch's *t*-test), with significance set at *P* < 0.05 and *P* < 0.01.

## 3. Results

### 3.1. Characteristics of Micropower Plasma and Production of Chemical Species

**Figure 2:**
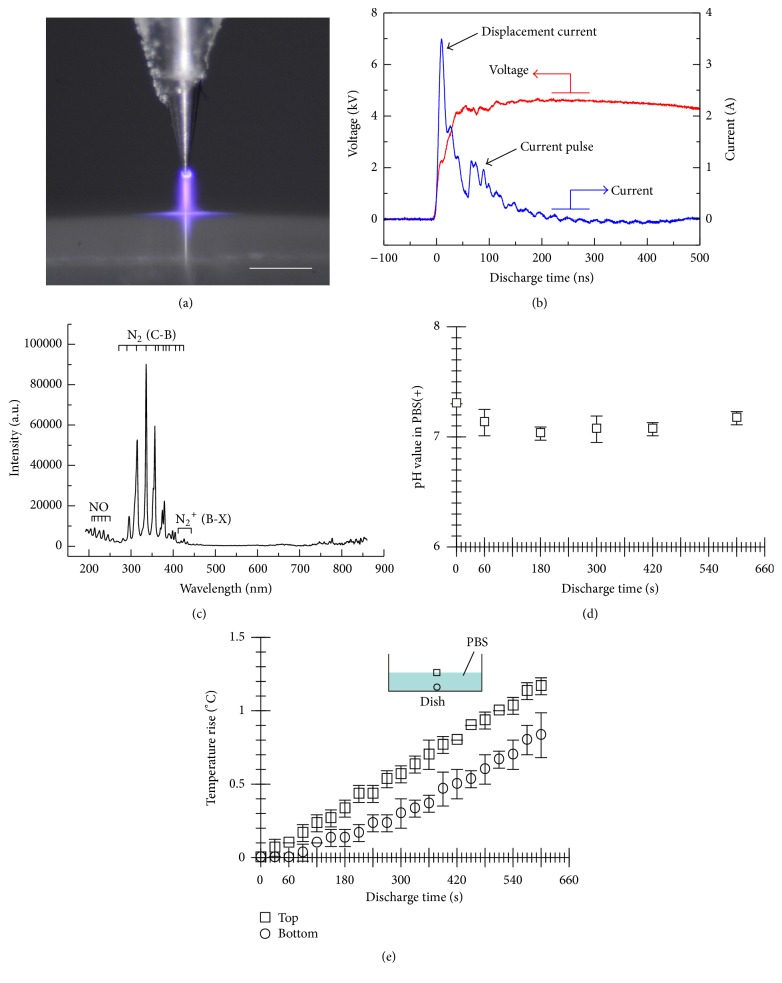
Characteristics of the micropower plasma. (a) Photograph of light emitted by the micropower plasma generated at the gas-PBS(+) interface. Bar is 500 *μ*m. (b) Waveforms of the applied voltage and discharge current. (c) Emission spectra of the plasma discharge at the gas-PBS(+) interface. (d) Changes in pH in the plasma-treated PBS(+) as a function of discharge time (*n* = 3, mean ± SD). (e) Changes in temperature of the plasma-treated PBS(+) with the discharge time (*n* = 3, mean ± SD).

Figure 2 shows characteristics of the micropower plasma generated at the gas-liquid interface. A photograph of plasma emission at the gas-liquid interface is shown in Figure 2(a). The applied voltage rose up to +5.5 kV with a rise time of 100 ns, and 3.5 A of displacement current and about 1.2 A of the current pulse were observed, as shown in typical waveforms of the applied voltage and the discharge current (Figure 2(b)). The power input *W* per cycle was 0.018 W, which was calculated from the following equation:

(1) $$\begin{array}{c} W=\frac{1}{T}\overset{T}{\underset{0}{\int }}v\left(\;t\;\right)i\left(\;t\;\right)dt. \end{array}$$

Here, *v*(*t*) and *i*(*t*) represent the applied voltage and the discharge current, respectively, and *T* represents the cycle length.

Figure 2(c) shows emission spectra of the plasma discharge at the gas-liquid interface. Emission peaks attributed to NO*γ* (222.2~272.2 nm), N_2_-SPS, and N_2_^+^-first negative system (FNS) (295.3~457.4 nm) were observed [16, 17]. Based on the observed emission spectra, the vibrational and rotational temperatures of N_2_ were calculated as 0.32 eV and 0.10 eV, respectively.

The change in pH in the PBS(+) as a function of plasma discharge time is shown in Figure 2(d). The pH in the PBS(+) decreased slightly, from 7.3 to 7.0, and was maintained about 7.0 over the 600 s discharge time. The temperature at the top surface of PBS(+) and the bottom surface in the dish rose monotonically with the discharge time as shown in Figure 2(e). The temperature rises were up to 1.2°C at the top surface of PBS(+) and 0.8°C at the bottom surface in the dish, respectively, by the discharge for 600 s. The temperature at the top surface of PBS and the bottom surface in the dish rose 0.5 and 0.2°C, respectively, by 300 s of the plasma discharge.

**Figure 3:**
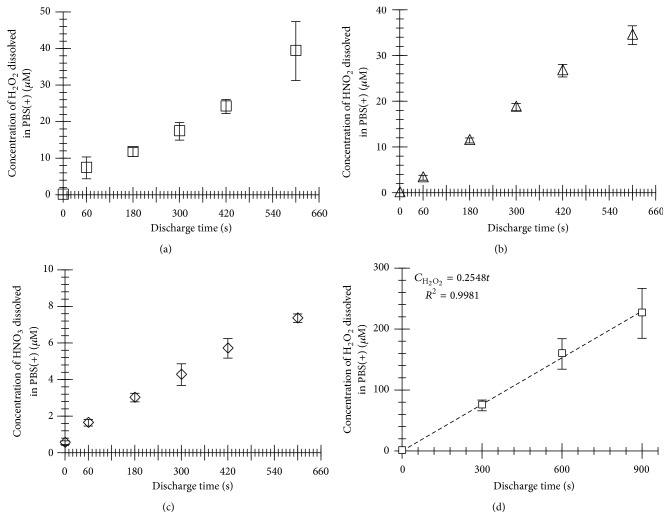
Concentrations of the dissolved H_2_O_2_ (a), HNO_2_ (b), and HNO_3_ (c) into the plasma-treated PBS(+) in a 35-mm diameter dish or the dissolved H_2_O_2_ (d) into PBS(+) in a 96-well plate, as a function of the discharge time (*n* = 3, mean ± SD).

Changes in the concentrations of H_2_O_2_, HNO_2_, and HNO_3 _dissolved into the PBS(+) in the 35-mm diameter dish with plasma discharge time are shown in Figures 3(a)–3(c). The concentrations of dissolved H_2_O_2_, HNO_2_, and HNO_3_ increased linearly during the plasma discharge for 600 s. Assuming the experiments using the cells cultured in 96-well plate, we also measured changes in the concentration of dissolved H_2_O_2_ with the discharge time for the PBS(+) in the 96-well plate, as shown in Figure 3(d). In the case of using the 96-well plate, the concentrations of dissolved H_2_O_2_ also increased linearly with the discharge time. Based on that result, we adjusted the concentration of H_2_O_2_ to which the cells were exposed.

### 3.2. Cell Viability, ROS in the Cells, and NF-*κ*B Activation after Treatment of Micropower Plasma

**Figure 4:**
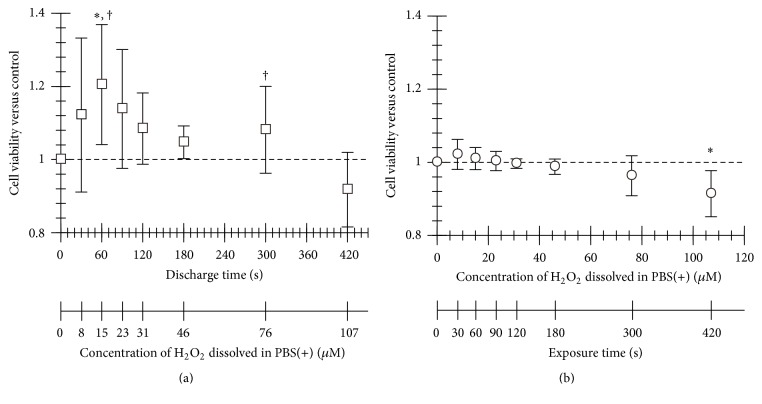
Effect of the micropower plasma treatment on proliferation in HUVECs. (a) Cell viability after the plasma treatment and 24-hour incubation (*n* = 3, mean ± SD). (b) Cell viability after the treatment of H_2_O_2_, with the concentration corresponding to the plasma treatment condition and 24-hour incubation (*n* = 3, mean ± SD). The viability of the cells shows a significant difference between the plasma- and H_2_O_2_-treated conditions (^†^ *P* < 0.05 versus H_2_O_2_-treated condition at the point with same time and concentration of H_2_O_2_). ^*∗*^ *P* < 0.05 versus the viability before the treatment (0 s or 0 *μ*M).

The plasma-generated H_2_O_2_ is, reportedly, a key factor to induce various responses to the plasma generated at an interface between gas and liquid phases [18]. Thus, we also compared the effects of plasma treatment on the cells with that of H_2_O_2_ treatment in the present study. As shown in Figure 4(a), the viability of the plasma-treated HUVECs increased and reached to the peak value by 60 s of the discharge time. Here, the concentration of H_2_O_2_ dissolved in PBS(+) was 15 *μ*M. At this point, the viability of the cells shows a significant difference between the plasma- and H_2_O_2_-treated conditions (*P* < 0.05, Figures 4(a) and 4(b)). The plasma-treated condition also resulted in significant increases in the cell viability, in comparison with that of the cells before treatment (*P* < 0.05, Figure 4(a)). For the discharge time longer than 60 s, the viability gradually decreased down to 0.87. As a control experiment, we exposed the cells to PBS(+) containing H_2_O_2_ with the concentration corresponding to that of the plasma-treated PBS(+). In contrast to the plasma-treated cells, although the viability of the cells exposed to H_2_O_2_ increased slightly for the conditions of low concentrated H_2_O_2_, it decreased monotonically, as shown in Figure 4(b).

**Figure 5:**
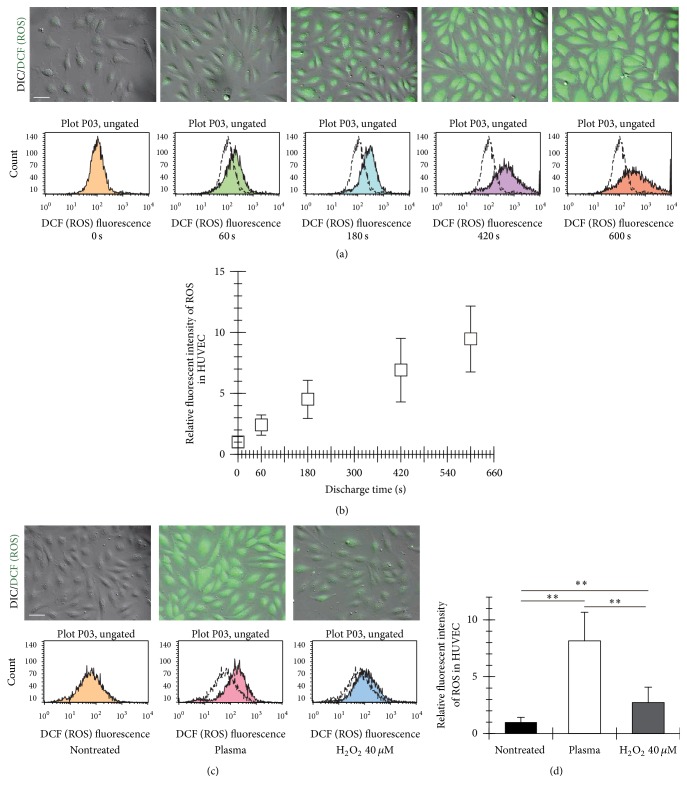
Fluorescence ROS intensity in HUVECs after the micropower plasma treatment. (a) Series of fluorescent images of ROS in the cells and representative histograms of ROS intensity from 5000 cells, measured using the flow cytometer after the plasma treatment and 15-min incubation in the MM. (b) ROS in the cells was expressed as the relative intensity. The basal value in the sample before the plasma treatment was taken as 1. The data shown were represented as mean ± SD (*n* = 3, 148 cells). (c) Representative fluorescent images of ROS in the cells treated with the micropower plasma and 40 *μ*M H_2_O_2_ for 600 s and representative histograms of ROS intensity from 5000 cells. (d) ROS in the cells treated with the micropower plasma or 40 *μ*M H_2_O_2_ for 600 s was expressed as the relative intensity. The basal value in the nontreated sample was taken as 1. The data shown were represented as mean ± SD (*n* = 3, 147 cells). The plasma treatment resulted in a significant increase in production of ROS in the cells (^*∗∗*^ *P* < 0.01 versus control and treatment of H_2_O_2_).

The ROS in the plasma-treated HUVECs linearly increased with the discharge time, as shown in Figures 5(a) and 5(b). The 9-fold increase in the fluorescence ROS intensity of the plasma-treated cells was found at 600 s of the discharge time, in comparison with the nontreated cells. The fluorescence intensity of ROS in the cells treated with the plasma discharge for 600 s was 3-fold higher than that under the condition exposed to H_2_O_2_, as indicated in Figures 5(c) and 5(d). The concentration of H_2_O_2_ was 40 *μ*M.

**Figure 6:**
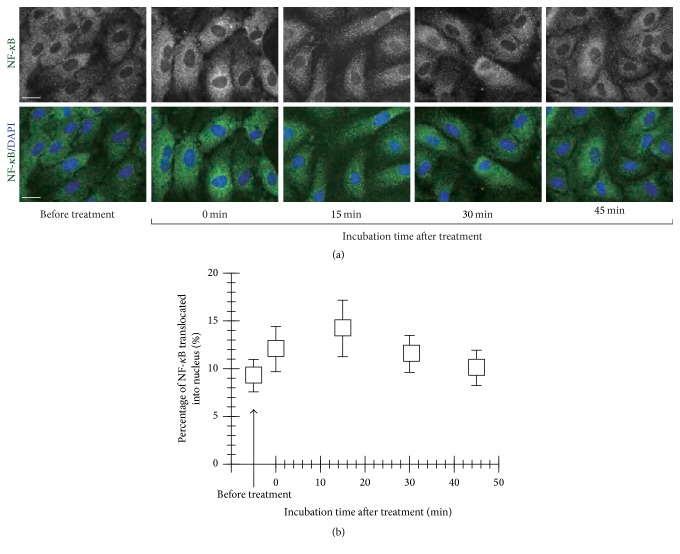
Activation of NF-*κ*B in HUVECs induced by the micropower plasma treatment. (a) Series of fluorescent images of NF-*κ*B in the cells after the plasma treatment for 300 s and incubation in the MM for 0, 15, 30, and 45 min. (b) Changes in percentage of NF-*κ*B translocated into cell nucleus with incubation time after the plasma treatment. The data shown were represented as mean ± SD for at least 50 cells.

**Figure 7:**
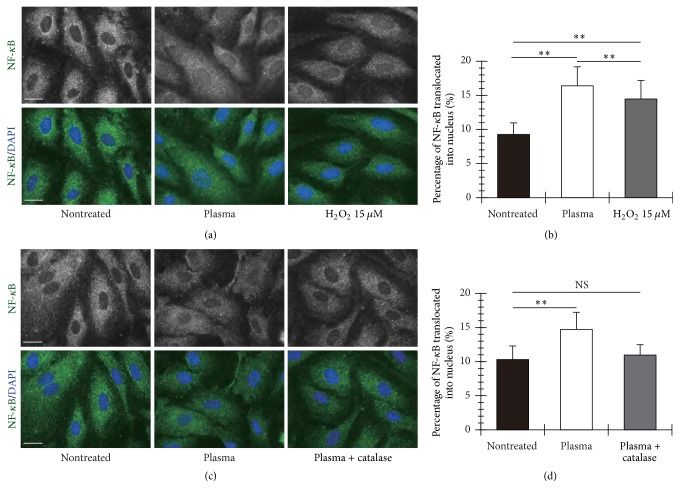
H_2_O_2_ generated by the micropower plasma is required for activation of NF-*κ*B in the HUVECs. (a) Percentage of nuclear translocation of NF-*κ*B in the cells treated with the micropower plasma or 15 *μ*M H_2_O_2_ for 300 s. The data shown were represented as mean ± SD for at least 55 cells. The plasma treatment resulted in a significant increase in the nuclear translocation of NF-*κ*B in the cells (^*∗∗*^ *P* < 0.01 versus control and treatment of H_2_O_2_). (b) Percentage of nuclear translocation of NF-*κ*B in the cells treated with the micropower plasma with/without 50 unit/mL catalase. The data shown were represented as mean ± SD for at least 57 cells. No difference in the percentage of nuclear translocation of NF-*κ*B was observed between the nontreated and the plasma-treated conditions when H_2_O_2_ was decomposed by using catalase.

NF-*κ*B is known to control cell death and survival decisions in cells. If the NF-*κ*B is activated, the activated NF-*κ*B translocates into the cell nucleus and results in the transcription of genes for cellular response such as cell death, survival, and growth [19]. Thus, focusing on translocation of NF-*κ*B into the nucleus, we can evaluate activation of NF-*κ*B indirectly. Based on the fluorescent images of NF-*κ*B p65 in the HUVECs for 0, 15, 30, and 45-min incubations after treatment of the plasma for 300 s (Figure 6(a)), we evaluated the translocation of NF-*κ*B p65 into the cell nucleus, as shown in Figure 6(b). Percentage of NF-*κ*B p65 translocation into the cell nucleus reached to the peak value (14%) by 15 min after treatment of the plasma, and then it decreased down to the level equivalent to before treatment. The concentration of H_2_O_2_ generated by the plasma discharge for 300 s was 15 *μ*M. Here, the nuclear translocation of NF-*κ*B p65 in the cells exposed to PBS(+) containing 15 *μ*M H_2_O_2_ for 300 s was confirmed to be lower than that in the plasma-treated cells (Figure 7(a)). It shows a significant difference between the plasma- and H_2_O_2_-treated conditions (Figure 7(b)). In the case of decomposition of H_2_O_2_ with 50 unit/mL catalase, the nuclear translocation of NF-*κ*B p65 in the cells induced by the plasma treatment was not confirmed as shown in Figures 7(c) and 7(d). These results suggests the plasma-generated H_2_O_2_ in PBS(+) was a main factor for the nuclear translocation of NF-*κ*B p65 in the plasma-treated HUVECs.

## 4. Discussion

### 4.1. Performance of the Micropower Plasma

In the present study, we used the micropower plasma with 0.018 W of the power input per cycle. The micropower plasma was able to generate small amount of the chemical species. The concentrations of the dissolved H_2_O_2_, HNO_2_, and HNO_3_ increased linearly during 600 s plasma discharge. The pH in PBS(+) showed little change after treatment of the plasma. The temperature rise in PBS(+) was up to 0.8°C at the bottom surfaces in the dish. This temperature rise is considered to have little influence on endothelial cells. This notion is supported by the report that the viability of HUVECs was not affected by heat stress at 39°C [20]. The micropower plasma can expose living cells to various reactive chemical species without harmful effects of heat and change in pH, in comparison with the plasma at the gas-liquid interface used in the previous studies [9–14].

Various chemical reactions are considered to occur during the plasma at gas-liquid interface [21, 22]. It is assumed that HNO_2 _and HNO_3_, which were detected in the plasma-treated PBS(+) in this study, were generated in a gas phase and then dissolved into the PBS(+). These chemical species were formed by reacting NO and NO_2_ with OH, as shown in the following reactions. NO and NO_2_ were generated by the plasma discharge in the gas phase. OH was generated by disassociation of H_2_O.

(2) NO2+OH+M⟶HNO3+M

(3) NO+OH+M⟶HNO2+M

Here, *M* indicates the third body, which is typically N_2_ or O_2_. On the other hand, H_2_O_2_ was produced by the following reaction between OH groups. The OH group was generated by the disassociation of H_2_O due to the plasma at the gas-liquid interface [23].

(4) $$\begin{array}{c} OH+OH⟶H_{2}O_{2} \end{array}$$

In addition, the micropower plasma might produce O_3_ according to the following reaction [24]. The oxygen atom is generated by the plasma discharge.

(5) $$\begin{array}{c} O+O_{2}+M⟶O_{3}+M \end{array}$$

### 4.2. Effects of Micropower Plasma on HUVECs

The results of the present study demonstrate that the short-time treatment of the micropower plasma enhances proliferation activity of HUVECs. The treatment of H_2_O_2_ with the concentration equivalent to that generated by the plasma, in contrast, does not enhance the cell proliferation. The ROS production and the NF-*κ*B activation due to the plasma treatment may play important roles for enhancement of the cell proliferation. This notion is supported by the findings that there were differences in the ROS production and NF-*κ*B activation, which was mainly induced by H_2_O_2_, in the cells between the plasma- and H_2_O_2_-treated conditions.

The fluorescence intensity of ROS in the cells treated with the plasma was much larger than that under the condition exposed to H_2_O_2_. Plasma is known to induce cell permeabilization [25]. The influx of the H_2_O_2_ generated by the plasma into the cells was, thus, increased due to permeabilization of the cell membrane for the treatment of plasma discharge. In addition, HNO_2_ and HNO_3_ generated by the plasma discharge are considered to have no small effect on increase in the ROS production in the cells by the plasma treatment.

Several studies have examined enhancement of proliferation in the vascular endothelial cells by nonthermal plasma [7, 8, 26]. Most of the mechanisms by which proliferation activity of the cells is enhanced are still unclear. The present study focused on the ROS production and the following NF-*κ*B activation after treatment of the plasma as the mechanism leading to the cell proliferation. NF-*κ*B is known to regulate many important cellular behaviors, such as inflammatory responses, cellular growth, and apoptosis [27, 28]. Reportedly, NF-*κ*B also regulates the expression of cyclin D1, which promotes cell cycle progression [29]. The activation of NF-*κ*B is caused by the ROS stimulation including H_2_O_2_ [30]. Increase in production of H_2_O_2_ by mitochondrion is known to enhance the activation of NF-*κ*B in the vascular endothelial cells [31]. These results reported in the previous studies can support our notion that the ROS production and the NF-*κ*B activation due to the plasma treatment may play important roles for enhancement of the cell proliferation.
